# A New Process of Preparing Rice Straw-Reinforced LLDPE Composite

**DOI:** 10.3390/polym14112243

**Published:** 2022-05-31

**Authors:** Huicheng Xu, Mengyuan Dun, Zhengqi Zhang, Lei Zhang, Weidong Shan, Weihong Wang

**Affiliations:** Key Lab of Bio-based Material Science & Technology of Education Ministry, Northeast Forestry University, Harbin 150040, China; hynner@163.com (H.X.); dunmengyuan0808@ahszu.edu.cn (M.D.); zhangzhengqi970617@163.com (Z.Z.); z1857910712@163.com (L.Z.); shanweidongnefu@163.com (W.S.)

**Keywords:** straw, linear low-density polyethylene film, composite, roll, mechanical properties, thermostability

## Abstract

To reduce the pollution resulting from discarding waste plastic film and burning straw, a new method of preparing straw-reinforced LLDPE composites was developed to utilize these wastes. The straws were first laid parallel on an LLDPE film and then rolled up. The rolls containing long straws were laid into a mat and then hot-pressed into a long straw composite board (the mass of straw accounted for 60%). Slope-cutting the straw, grinding the straw, and twisting the roll were designed to improve the physical and mechanical properties of long straw composites. Among them, slope-cutting the straw combined with twisting the roll provided the best properties. Compared to the extruded straw particle composite, the composite prepared with the new method improved the tensile strength, bending strength, impact strength, and water resistance by 358%, 151%, 416%, and 81%, respectively. Slope-cutting exposed more inner surface at the end of the straw. Scanning electron microscope observations showed that the straw inner surface was more tightly bonded with the LLDPE matrix than the outer surface. Meanwhile, the integrity of the straw was retained as much as possible, and thus greatly improved the performance of the resulting composites. Dynamic mechanical analysis, differential scanning calorimetry, and thermogravimetric analysis show that the viscous deformation of the composites prepared by the new method was reduced and the rigidity was increased, and the combination of straw and LLDPE forms a dense composite material with good interfacial bonding. It greatly slowed down the degree of its pyrolysis.

## 1. Introduction

Crop straw is a natural, renewable, reusable, and environmentally friendly material. The world produces a large amount of straw every year. For example, China’s straw output in 2019 was about 1 billion tons [1]. However, only a small part of these straws is used, and most of them are abandoned or incinerated, which not only causes serious pollution to the environment but also wastes biomass resources. In addition, polyolefin film is widely used in agricultural production, of which LLDPE film accounts for a large proportion. LLDPE has the advantages of high weather resistance, tear resistance, and chip toughness, but its service life is short (3–4 months). Therefore, it is also imperative to develop effective methods to utilize recycled agricultural film. 

Combining crop straw fibers with thermoplastic polymer to prepare composite materials with good performance [2,3,4,5,6,7] is one of the effective ways to utilize these waste resources. There have been some studies on the preparation of straw–plastic composites (also belong to wood–plastic composite materials). For example, Lin et al. [8] prepared straw particle-reinforced high-density polyethylene composites by injection, and Zhang et al. [9] prepared composites using wheat straw powder and recycled polyethylene as raw materials. Both studies confirmed that straw increased the flexural and tensile strength of the composites. The most often used plastic matrix in the present composite is polyolefin resin, such as polyethylene [10,11] and polypropylene [12]. To improve the compatibility between straw and plastic, chemical methods are usually employed. Yiga et al. [13] treated rice husk with sodium hydroxide. Chen et al. [14] studied the effects of the silane coupling agent KH570 and nano-TiO_2_ modification on the properties of wheat straw/polylactic acid (PLA) composites, while Liao et al. [15] evaluated the effect of nano-TiO_2_ with different particle sizes on the properties of wheat straw powder/PLA composites. These chemical methods improve the performance of composites but have potential problems such as polluting the environment and increasing production costs. In the above-mentioned research and current industry production, most of the straw–plastic composite materials are prepared by first breaking the straw into particles and then mixing it with plastic particles. This process results in the problem of wasting raw straw materials, and damaging straw fibers. In addition, the straw particles have a small bulk density, which makes it difficult to uniformly mix with plastic particles that have higher densities.

To prepare biofiber/plastic composites with high mechanical properties, our research group developed a new method that maintained the original slender form of straw and hemp fiber in the composites. We used plastic film as a carrier, wrapping straw or long hemp fiber into rolls and then hot-pressing them [16,17]. It is not necessary to cut straws or other long fibers into particles in this new method. The mechanical properties of the resulting composites were significantly improved compared with the traditional particle/plastic composites. The physical treatment of straw or long fibers has advantages in environmental protection and low investment costs, which is worthy of further study.

Straw–plastic composite materials can be used in the packaging materials industry, vehicle engineering [18,19], and daily necessities’ production and other industries [20]. In this paper, we continue to optimize the new technology to improve the performance of straw–plastic composites and analyze the principle of this improvement. Firstly, the straws were rolled together with LLDPE film, and then the rolls were arranged in parallel onto a mat and hot-pressed into long straw composites.

Previous studies have shown that the inner surface of straw has stronger interfacial bonding force than the outer surface [21,22,23,24,25]. The best mechanical properties can generally be obtained for composites when the fiber is aligned parallel to the direction of the applied load [26,27,28]. At all testing temperatures, the composites containing larger-sized WFs had greater creep resistance than those containing smaller-sized fibers. The former has a larger aspect ratio [29]. The interfacial bonding between fibers and resins is another important factor affecting the mechanical properties of composites. However, for plant fiber-reinforced thermoplastic composites, poor interfacial bonding between hydrophilic fibers and the matrix can seriously affect the mechanical properties of the composites [30]. For bonding to occur, fiber and matrix must be brought into intimate contact [31].

Therefore, the present study adopted two methods (slope-cutting and grinding the straw) to expose more of the inner surface of the straw tube. Furthermore, the rolls were twisted to enhance the straw pullout resistance. Via these physical treatments, the physical and mechanical properties of the straw–plastic composite were improved. This research promotes the utilization of straw biomass and recycled plastic film resources. 

## 2. Materials and Methods

### 2.1. Materials

Annual straw, purchased from Yanshou County, Harbin City, is about 70–90 cm long, has a bulk density of 0.035g/cm^3^, tensile strength of 44.36 MPa without knots and 27.89 MPa with knots, and the internode tensile elastic modulus is 1.438 GPa. The LLDPE film was purchased from Shanghai Runwen Packaging Materials Co., Ltd., with a thickness of 0.01 mm, a density of 0.923 g/cm^3^, tensile strength of 10.0 MPa, melting enthalpy of 113.1 J/g, a tensile modulus of elasticity of 200 MPa, and a melting temperature of 122.90 °C. Maleic anhydride-grafted polyethylene (MAPE) powder was purchased from Nantong Rizhisheng Company, with a graft ratio of 0.9% and a density of 0.92 g/cm^3^.

### 2.2. Preparation of Straw/LLDPE Composite

**Figure 1:**
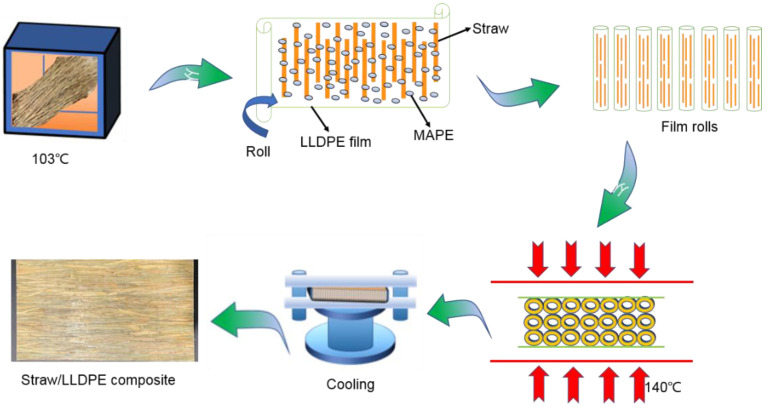
Process of preparing the straw composite.

**Figure 2:**
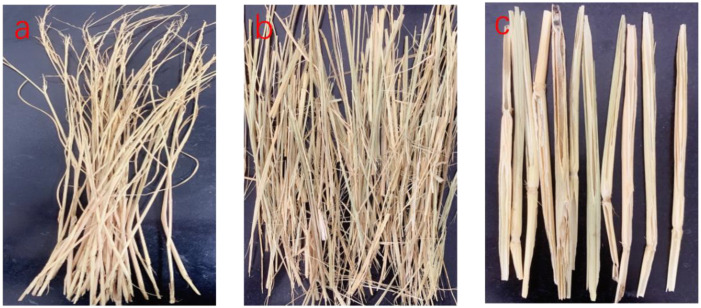
Straw (**a**), grinded straw (**b**), and slope-cut straw (**c**).

The preparation process of straw composites is shown in Figure 1. First, the straw was dried to a moisture content of less than 3%. The long straws (Figure 2a) were subjected to grinding or slope-cutting, respectively. The length of the grinded straw was 15–20 cm (Figure 2b). The slope-cut straw (Figure 2c) was 15 cm-long, and the cutting angle at the end of the straw was less than 60°. The treated or untreated straws were laid in parallel on the LLDPE film, the MAPE powder was evenly dispersed on it, and then the LLDPE film and the straw were rolled up together to form a long film roll (each roll was 430 mm in length). Some rolls were applied with two twists (one twist is 360°). The twisted or untwisted film rolls were laid parallel into a mat in a mold, and then placed in a hot press. The mat was pre-pressed for 4 minutes (at a pressure of 0.5 MPa) and hot-pressed for 6 minutes (at a pressure of 10 MPa) at a temperature of 140 °C. After cooling to room temperature, the long straw composite board was obtained. The size of the board was 430 mm × 220 mm × 8 mm. The density of the composites was 0.80g/cm^3^. The mass of straw accounted for 60%, LLDPE accounted for 37%, and MAPE accounted for 3%.

As a control, the straw particle composite was prepared according to the conventional extrusion process. First, the dried straw granules (20–40 mesh, 830–630 micron.), LLDPE granules, and MAPE powder are mixed in a high-speed mixer, first at a low speed for 10 s, and then at a high speed for 5 minutes. Then, the mixture was sent to a twin-screw extruder for melt compounding, and the processing temperature of each section of the twin-screw extruder was set to 135 °C/140 °C/140 °C/135 °C. The mixture was cooled to room temperature and broken into pellets. The pellets were fed into a single-screw extruder and extruded into a straw pellet/LLDPE composite pipe with a cross-sectional dimension of 40 mm × 4 mm. The density was 1.10g/cm^3^. The processing temperature of each stage of the single-screw extruder was set at 135 °C/140 °C/140 °C/135 °C.

### 2.3. Characterization of the Straw/LLDPE Composite

#### 2.3.1. Mechanistic Property Tests

An electronic universal testing machine (CMT5504 universal testing machine, USA Sterling Industrial Straws Co., Ltd., Wuhan, China) was employed to test the tensile property of the composites. The dumbbell-shaped pieces (length 165 mm, narrow part width of 13 mm, thickness of 8 mm) were prepared for tensile testing. The gauge length was 50 mm, and the loading speed was 5 mm/min. At least 6 specimens were tested in each group.

The three-point bending property of the composites was tested by the same machine. The bending test speed was 10 mm/min, the span was 128 mm, and the size of the specimen was 160 mm × 15 mm × 8 mm. At least 6 specimens were tested in each group.

The impact strength of the composites was tested by a simply supported beam impact testing machine (JC-5 pendulum impact testing machine, Chengde Precision Testing Machine Co., Ltd., Wuhan, China). The size of the unnotched specimen was 160 mm × 15 mm × 8 mm. The impact energy was 5 J, the pendulum velocity was 3.8 m/s, and at least 6 specimens were tested in each group.

Origin 2018 software (Origin Lab Co., Wuhan, China) was used to plot the data. Statistical analysis was performed using SPSS 20.0 software (SPSS Inc., Wuhan, China). One-way analysis of variance (ANOVA) was used to test for a significant difference between different groups. *p* < 0.5 signifies significant differences in data between different groups.

#### 2.3.2. Water Resistance Performance

The specimens with a size of 50 mm × 50 mm × 8 mm were immersed in water at (20 ± 1) °C. After soaking for 24 h ± 10 min, the specimens were taken out and their surface water was wiped off. The thickness of the specimen was quickly measured, and the expansion rate in thickness due to water absorption was calculated based on the original size before soaking. At least 6 specimens were tested in each group.

#### 2.3.3. Micro-Morphological Observation 

The internal structure of the straw and composites was observed by a scanning electron microscope (SEM, Quanta 200F field emission scanning electron microscope, FEI Company, Waltham, MA, USA). The specimen was frozen in liquid nitrogen and then broken. The cross-section of the specimen was sprayed with gold, and then observed and photographed in the SEM at an accelerating voltage of 12.5 kV. 

#### 2.3.4. Differential Scanning Calorimetry (DSC)

The composite samples were analyzed by a differential scanning calorimeter (DSC-Q20 Differential Scanning Calorimeter, TA Company, John Hancock Tower Boston, MAUSA). At a rate of 10 °C/min, the temperature was lowered from room temperature to −80 °C, and then heated to 200 °C. The samples were granular, about 2–3 mm in diameter. Samples were taken at multiple locations on the composite. The relationship between the enthalpy change and the temperature of the sample due to the change of physical and chemical properties was measured. The entire testing process was performed under a nitrogen atmosphere.

#### 2.3.5. Thermogravimetric Analysis (TG)

A thermogravimetric analyzer (STA 6000-SQ8 type thermogravimetric analyzer, PerkinElmer Co., Ltd. (Waltham, MA, USA) was used to measure the mass change of the sample with increasing temperature. The samples were heated from room temperature to 600 °C at a heating rate of 10 °C/min. The samples were granular, about 2–3 mm in diameter. The entire testing process was performed under a nitrogen atmosphere.

#### 2.3.6. Dynamic Mechanical Analysis (DMA)

A dynamic mechanical analyzer (DMA-242C dynamic mechanical analyzer, TA Instruments-Waters LLC, New Castle, DE, USA) was used to measure the variation of the mechanical properties of the samples with temperature. The test temperature range was −35∼120 °C, the heating rate was 3 °C/min, and the size of the sample was 35 mm-long, 14 mm-wide, and 4 mm-thick. The single cantilever beam test mode was adopted, and the deformation frequency range was 0.1–100 Hz. The whole test process was protected by N_2_ atmosphere.

## 3. Results

### 3.1. Mechanical Properties of Long Straw/LLDPE Composite

**Figure 3:**
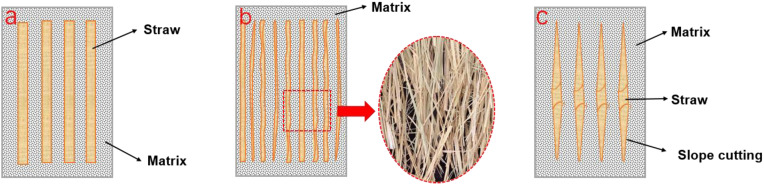
Distribution of straw units in the LLDPE matrix: straw (**a**), grinded straw (**b**), and slope-cut straw (**c**).

Compared to the straw particle composite prepared by the traditional extrusion method, the long straw composite prepared by the new method significantly improved the properties of tensile, bending, and impact resistance with an increase of more than 200%. Among them, compared to untreated rice straw (Figure 3a), slope-cutting the straw, combined with twisting the film roll, played the most positive role. It has been reported that the silicon on the surface of straw adversely affects its bonding with the matrix, while the silicon content on the inner surface of straw is much lower than that on the outer surface [32,33]. Although grinding the straw into strips facilitated the penetration of the LLDPE matrix, it destroyed the integrity (Figure 3b) and reduced the strength of the straw itself. Under the comprehensive effect, the improvement of the mechanical properties of grinded long straw composites was not as high as that of slope-cut straw composites. Thus, maintaining the structural integrity of straw is important in improving the mechanical properties of straw composites.

By slope-cutting, the straw exposed its inner cavity surface at both ends within a length of about 60 mm (Figure 3c). During the hot-pressing process, the molten LLDPE matrix easily penetrated into the cavity of the straw and bonded with more inner surface, thereby improving the interfacial bonding strength between the straw and the matrix.

Applying twisting treatment to the film roll changed the straw from the parallel untight arrangement to the spiral winding state. Straw and fiber bundles were wound together and the friction between them increased. This structure improved the LLDPE matrix’ s reinforcement. Therefore, twisting the film roll improved the flexural and tensile strength of the long straw composite, and the improvement in modulus was more obvious. When subjected to impact stress, this good interfacial bonding and longer failure path made the composite consume more energy, thus improving the impact resistance of long straw composites. 

The theoretical stiffness value of the straw composite can be simply calculated according to the rule of the following mixture (Equation (1)):

(1) $$E_{c}=E_{f}V_{f}+E_{m}\left(1-V_{f}\right)$$

The theoretically predicted tensile modulus value (*E_c_*) is 1.07 GPa based on the data in Section 2.1 and Section 2.2 (the fiber tensile modulus (*E_f_*) was 1.44 GPa, the matrix tensile elastic modulus (*E_m_*) was 0.20 GPa, the density of the composites was 0.80g/cm^3^, the density of the LLDPE was 0.923g/cm^3^, and the calculated fiber volume fraction (*V_f_*) was 70%). The experimental value was much higher than the calculated value. The reason might be that the rice straw was compacted, and the modulus of the dense straw fiber was higher than that of intact straw.

**Figure 4:**
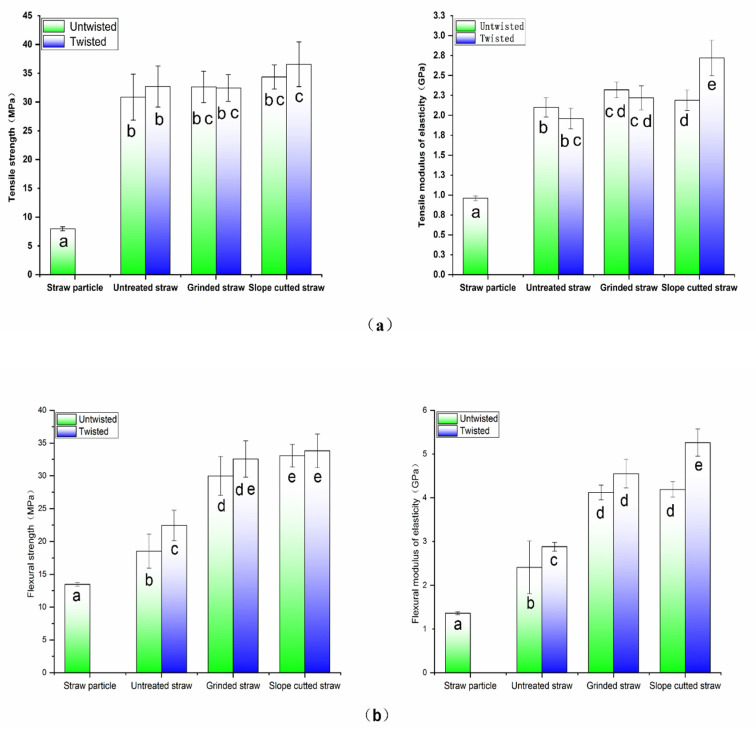

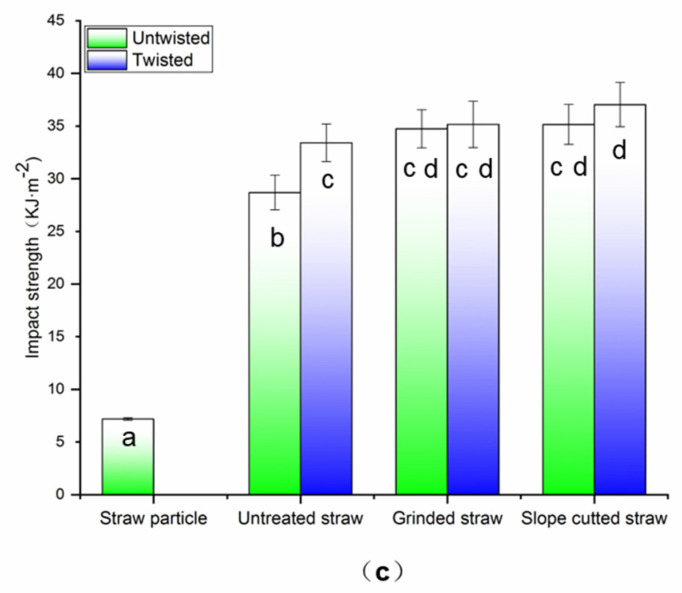
Mechanical properties of straw/LLDPE composites with different shapes (the same letters at the top of the bar chart indicate no significant difference): tensile property (**a**), flexural property (**b**), and impact strength (**c**).

Similar to the calculation of the modulus, the predicted tensile strength according to the mixing rule was 34.05 MPa, which is approximately equal to the values obtained from the practical test, except for the slope-cut straw/LLDPE composite (Figure 4a), which was higher.

### 3.2. The Water Resistance of Long Straw Composites

**Figure 5:**
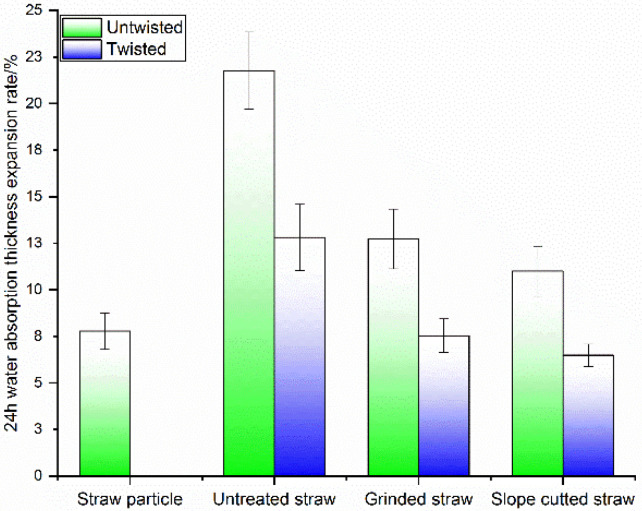
Thickness expansion ratio diagram of straw pellets and long straw-reinforced LLDPE composites after 24 h water absorption.

Compared with the straw particle composite, the long straw without pretreatment presented a significant increase in the thickness expansion rate for the composite due to water absorption (Figure 5). The reason should be that the inner surface of the long straw cavity lacked matrix coverage, and the exposed hydroxyl groups led to higher water absorption.

The thickness expansion rate could be significantly reduced by slope-cutting and grinding straw. Both grinding and slope-cutting helped the molten LLDPE matrix penetrate into straw fiber bundles and coat the straw inner surface, respectively. Thus, the naked area of straw without matrix covering was reduced, and water absorption was prevented. Twisting made the composite structure compact, which is an important factor in further reducing water absorption and inhibiting expansion deformation. 

### 3.3. Microstructure of Long Straw/LLDPE Composites

**Figure 6:**
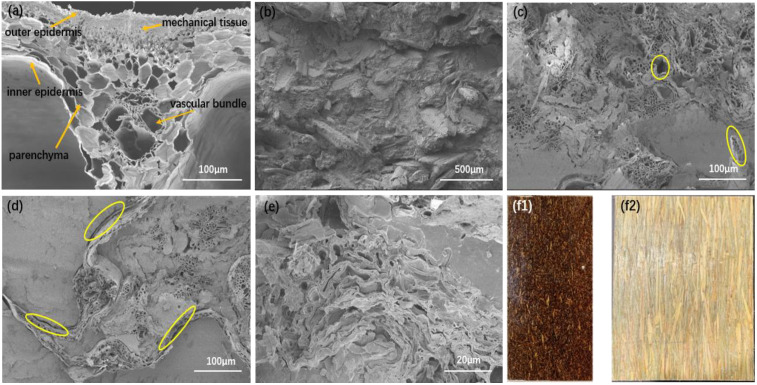
Structure of the straw and straw/LLDPE composite: cross-section of straw stalk (**a**), extruded straw particle composite (**b**), hot-pressed untreated straw composite (**c**), hot-pressed grinded straw composite (**d**), hot-pressed slope-cut straw composite (**e**), and appearance of extruded straw particle composite (**f1**) and hot-pressed straw composite (**f2**).

The microstructure of the straw composite was observed by using scanning electron microscopy (Figure 6a–e). On the cross-section of straw, epidermal tissue, mechanical tissue, parenchyma tissue, and vascular bundles were found [34,35,36], showing a large number of cell lumen pores (Figure 6a). In the extruded straw particle composite (Figure 6b), the fibers were randomly distributed in and closely combined with the LLDPE matrix.

In the long straw composite prepared from untreated straw, more oval or circular pores were exposed (Figure 6c). This would be the cavities of the straw and the parenchyma cells which were not filled by the matrix. It indicates the difficult penetration of LLDPE. The outside of the straw was surrounded with LLDPE, and the pores were left empty when the straws were pulled out or broken. 

For the grinded straw-reinforced LLDPE, the number of cavities in the composites was reduced. The broken cross-section showed a layer structure because of the existing straw strip. Both sides of the strips contacted the LLDPE matrix (Figure 6d), and the outside of the strips did not bond well with LLDPE, showing gaps. There were also pores left empty when the strips were pulled out. 

Figure 6e shows the composite sample prepared from slope-cut straw. It contains the position of slope-cutting. The parenchyma cells on the inner straw surface were exposed, which made the straw wall tightly bond with the LLDPE matrix. This indicates that the stress could be effectively transferred when the resulting composite is subjected to loading [16] and the straw could effectively strengthen LLDEP. 

In terms of appearance, the extruded particle composite was darker in color (Figure 6f_1_,f_2_) compared to the long straw composite. The reason is that higher shearing force and heating damage occurred during extruding. In addition, small straw particles have lower thermal resistance compared to long straws [30]. Friction, shear, and other forces caused more serious degradation and reduced the mechanical properties of the composite. 

### 3.4. Thermal Properties of Long Straw/LLDPE Composites

The crystallinity of LLDPE was calculated according to Equation (2):

(2) $$X_{c}=\frac{∆H_{f\;(m_{c∕m_{LLDPE}})}}{∆H_{f}^{0}}$$

where ∆*H_f_* denotes the melting enthalpy, mc denotes the mass of the sample, mLLDPE denotes the mass of LLDPE in the sample, and $∆H_{f}^{0}$ denotes the melting enthalpy of LLDPE with 100% crystallization, 293 J/g [37].

**Figure 7:**
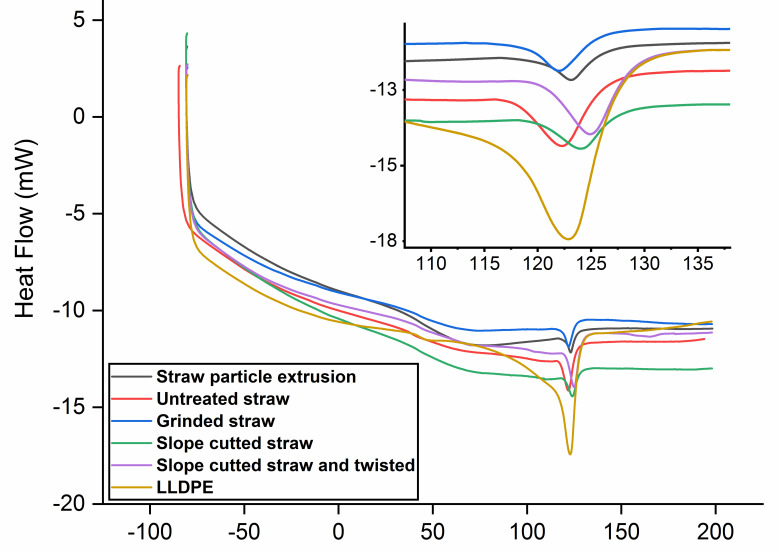
DSC thermograms of straw/LLDPE composites.

The data in Table 1 show that there was no significant difference in melting temperature among the five composite samples. However, the melting peaks of the long straw composites were wider and deeper than those of the extruded composite (Figure 7). As shown in Table 1, the former composites had a much higher melting enthalpy than the latter. This indicates that the long fibers have greater resistance to the melting of LLDPE, and so more heat needs to be absorbed during the melting process. The melting enthalpy of the composite is related to its crystallinity. The higher the crystallinity, the higher the heat required to melt the composite material; that is, the higher the melting enthalpy [38]. In Table 1, the long straw composite shows higher crystallinity, which should be the reason for its higher melting enthalpy. The reason for the higher crystallinity might be that the long straw composite prepared by the hot-pressing method experienced a slower cooling process than the extruded straw particle composite. Another possible reason for the higher melting enthalpy is that the hot-pressed composite has a lower density (0.85 g/cm^3^), leading to lower heat conduction than the extruded composite (1.1 g/cm^3^).

**Figure 8:**
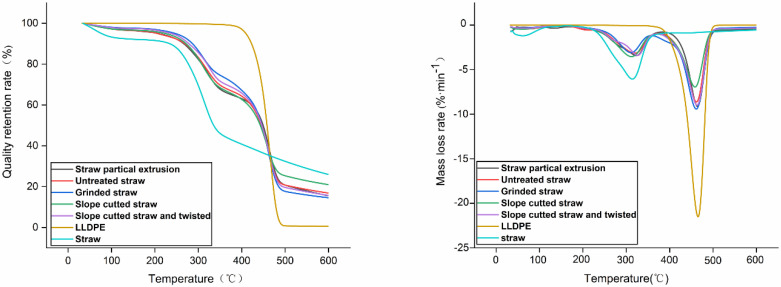
TG curve of straw, LLDPE, and straw/LLDPE composite.

As shown in Figure 8, LLDPE degraded rapidly in the range of 400 to 500 °C; in contrast, straw started to lose mass earlier and faster, and the main thermal degradation stage was between 180 and 430 °C.

The mass loss process of all the straw composites under the action of heat is similar, which is divided into four stages. At the first stage of weight loss, which occurred at room temperature to 100 °C, the straw underwent significant changes, however the composites were slightly degraded. The second stage occurred between 150 and 230 °C, in which both the straw and the composites changed slightly until the third stage began. The third stage was mainly caused by the decomposition of cellulose in straw [38]. The fourth stage of weight loss occurred between 350 and 500 °C, and was mainly caused by the degradation of LLDPE. Under 400 °C, the thermal degradation of straw composites is dominated by the decomposition of straw components; above 400 °C, LLDPE is the main factor. The combination of straw and LLDPE formed a dense composite material, and the good interfacial bonding greatly slowed down its pyrolysis degree. The straw, which was grinded, was better covered by the plastic matrix (Figure 6d), thus showing slightly stronger thermal stability than other long straws.

The thermal stability of extruded composites was lower. This is because in the extrusion process, the long straw was damaged by shear force and friction, resulting in a reduction in size. The distribution of short fibers in the matrix was uneven and discontinuous, which is prone to degradation under high-temperature conditions, resulting in the reduced thermal resistance of the composites. The long straw can not only maintain a high degree of orientation but can also better resist mechanical damage. Compared to straw particles, the intact straw absorbed and stored more heat, thereby improving the thermal stability of the resulting composite material. 

### 3.5. The Dynamic Mechanical Properties of Long Straw/LLDPE Composites

**Figure 9:**
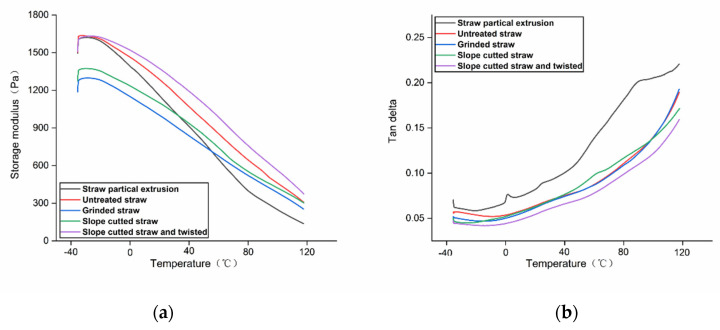
DMA thermogram of straw/LLDPE composites. (**a**)Storage modulus; (**b**)Tan data.

Dynamic mechanical analysis also showed that the enhancement of long straw was superior (Figure 9a). Compared with the straw particle composite, the long straw composites had a higher storage modulus at both low and high temperatures, indicating that the intact straw increased the service temperature of the composite. Other studies proved that the better the bonding state between plant fibers and thermoplastic resins, the higher the storage modulus and the superior mechanical properties [30,31]. The long straw composite obtained by slope-cutting and twisting treatment showed the highest storage modulus at room temperature, which is consistent with the measured static mechanical properties (Figure 4). At temperatures above 40 °C, it was still higher than the composites prepared by other processes (Figure 9a). The main reason could be that the slope-cut straw was relatively intact, so its own strength and heat resistance were better than that of the grinded material. Another reason could be that the slope-cutting improved the interface bonding performance of the composite.

When the slope-cut and twisted long straw composite was loaded, the energy loss caused by viscous deformation was relatively small. During the stress deformation process, the good interfacial bonding and compact structure inhibit the relative sliding of LLDPE molecular chains, enhancing the mechanical internal friction of the composite. While the particles of the straw composite showed higher viscous deformation and better flexibility, the combination of slope-cutting and twisting made the composite less viscous and more rigid, so it had the lowest loss factor (Figure 9b). 

## 4. Conclusions

In this study, a new process for preparing straw-reinforcing thermoplastic composites was developed. The long straw composites were prepared by wrapping the straw with LLDPE film and using hot-pressing. The effects of straw slope-cutting, straw grinding, and film roll twisting on the properties of the composites were discussed. The following conclusions were obtained based on the performance test and analysis.

The slope-cutting and grinding enabled the LLDPE matrix to penetrate into the straw tube, and thus increased the bonding area of the composites. Twisting made the straws have more contact with each other and with LLDPE. These factors improved the physical and mechanical properties of straw composites.

The developed method presented the long straw/LLDPE composites with high tensile strength, impact strength, flexural strength, and better water resistance, with values of 36.56 MPa, 37.03 kJ/m^2^, 33.82 MPa, and 6.48%, respectively. These are significantly better results than those of the traditional extruded straw particle/LLDPE composites. The latter’s tensile strength, impact strength, bending strength, and water resistance were 7.98 MPa, 7.18 kJ/m^2^, 13.47 MPa, and 11.73%, respectively. In addition, the intact rice stalks in the composites can absorb and store more heat, thereby improving the thermal stability of the composites. The thermal stability and service temperature of long straw composites were also improved.

The new process developed here will help to prepare biofiber composites with high performance.

## Figures and Tables

**Table 1:** Crystallinity, melting enthalpy, and melting temperature of straw/LLDPE composites.

Sample	X_c_ (%)	T_m_ (°C)	∆H_f_ (J/g)
Straw particle extrusion	4.81	123.15	4.15
Untreated straw	8.92	122.87	10.40
Grinded straw	8.20	122.01	8.89
Slope-cut straw	7.53	124.11	8.17
Slope-cut and twisted straw	12.5	124.99	13.52

X_c_—crystallinity; T_m_—melting temperature; ∆H_f_—melting enthalpy.
